# 血清白蛋白及尿素氮水平与肺癌不同临床病理特征和预后的关系

**DOI:** 10.3779/j.issn.1009-3419.2017.03.06

**Published:** 2017-03-20

**Authors:** 亚伦 李, 镭 李, 立 张, 为民 李

**Affiliations:** 610041 成都，四川大学华西医院呼吸内科 Department of Respiratory Medicine, West China Hospital, Sichuan University, Chengdu 610041, China

**Keywords:** 肺肿物, 血清白蛋白, 尿素氮, 预后, Lung neoplasms, Albumin, Urea, Prognosis

## Abstract

**背景与目的:**

肺癌作为全球男女性中致死率最高的肿瘤之一，严重威胁着人类的健康。本研究旨在分析肺癌患者血清白蛋白及尿素氮水平与临床病理特征及生存期的关系。

**方法:**

收集2008年1月-2013年12月四川大学华西医院病理确诊原发性肺癌患者1, 098例，检测患者血清中白蛋白及尿素氮水平，按检测水平是否正常分为阳性组及阴性组，分别分析白蛋白及尿素氮水平与肺癌临床病理特征及生存期的相关性。

**结果:**

1, 098例患者的年龄、性别、病理学分型、肝转移、胸膜转移与血清白蛋白水平差异有统计学意义（*P*＜0.05）；312例鳞癌患者的年龄、肺内转移以及612例腺癌患者的年龄、性别、临床分期、胸膜转移与白蛋白水平差异有统计学意义（*P*＜0.05）。尿素水平与各临床特征均无统计学意义。不同病理学类型的肺癌患者血清白蛋白水平在阴性组与阳性组中位生存时间分别为鳞癌36个月和19个月，腺癌35个月和15个月，前者中位生存期明显高于后者。尿素氮水平与肺癌患者生存期无相关性。

**结论:**

多种影响因素与血清白蛋白阳性程度相关，血清白蛋白水平与肺癌的生存相关，其是肺癌患者预测预后的一个指标。

## Abstract (en)

**Background and objective:**

Lung cancer is the most common malignancy and is the leading cause of cancer-related death worldwide. Thus, this disease severely threatens human health. This study aims to identify the relationships between serum albumin and urea level and the clinical pathological characteristics and survival time in patients with lung cancer.

**Methods:**

A total of 1, 098 patients with lung cancer were diagnosed by pathology and tested the serum albumin and urea level in West China Hospital of Sichuan University during January 2008 to December 2013. According to the levels of albumin and urea, patients were divided into the normal level group(negative group) and abnormal level group(positive group). The differences of patients' clinical pathological characteristics and survival time in the two groups were analyzed.

**Results:**

Differences in age, sex, histological classification, liver metastasis and pleural metastasis were statistically significant between the two groups of serum albumin(*P* < 0.05). Differences in age, intrapulmonary metastasis of 312 patients of squamous cell carcinoma and differences in age, sex, stages, pleural metastasis of 612 patients of adenocarcinoma between the two groups of serum albumin(*P* < 0.05). There were no significant differences between the two groups of urea. In different histological classification between the two groups of serum albumin, the median survival period of squamous cell carcinoma was 36 months and 19 monthes, adenocarcinoma was 35 months and 15 monthes, the abnormal group were all significantly lower than those in the normal group. The median survival period was no significant difference between the two groups of urea.

**Conclusion:**

The level of serum albumin is an important indicator for prognosis.

目前肺癌是全球发病率及致死率最高的肿瘤之一，非小细胞肺癌（non-small cell lung cancer, NSCLC）约占肺癌的80%，包括腺癌、鳞癌和大细胞癌；小细胞肺癌（small cell lung cancer, SCLC）约占20%，其恶性程度高，发展迅速，预后更差。尽管近些年治疗手段不断发展，但其5年生存率仅为17%^[1]^。世界卫生组织（World Health Organization, WHO）预测中国到2025年时每年新发肺癌病例将超过100万，成为世界肺癌第一大国^[2]^。

肺癌患者血清标志物监测对肺癌的诊断、治疗及预后有重要的临床意义。随着科技的进步，目前对肿瘤标志物的研究进展很快，临床多采用肿瘤标志物的联合检测。但在临床工作中，临床医师对血清中部分生化指标并不够重视。生化指标中血清白蛋白及尿素氮的测定值，可能会反应肺癌患者的预后。白蛋白主要在肝脏合成，其半衰期长，在体内广泛分布，以阻止体内营养状态的迅速改变。肺癌患者，尤其是疾病进展的患者，由于肿瘤代谢增强，能量消耗大，多数患者食欲下降，食物摄入量减少，白蛋白合成减少等因素，导致血清白蛋白水平下降。因此我们推测白蛋白水平不仅提示患者的营养水平，也和患者的预后有关。血清尿素氮是反应肾功能的主要指标之一，也是人体蛋白质代谢的主要终末产物。我们推测，血清尿素氮是否也和肺癌患者的预后等有相关性。因此本研究分析血清标志物中白蛋白及尿素氮指标与肺癌患者的临床特征及生存预后的差异。

## 1 资料与方法

### 1.1 研究对象

研究对象为2008年1月-2013年12月四川大学华西医院确诊原发性肺癌患者1, 098例，所有患者均随访24个月-36个月。纳入标准为初诊为原发性肺癌患者，所有患者均经病理学证实。排除标准为排除无病理学依据，临床资料不完整患者；以及合并其他肿瘤或肺癌复治的患者。收集资料包括肺癌患者年龄（＜45岁、45岁-60岁、＞60岁）、性别（男性、女性）、病理学分型（鳞癌、腺癌、小细胞癌）、临床分期（Ⅰ期、Ⅱ期、Ⅲ期、Ⅳ期）、吸烟史、淋巴结转移及远程转移（脑、骨、肝、肾上腺、肺内、胸膜、纵隔）情况。临床分期根据第七版美国癌症联合委员会（American Joint Committee on Cancer, AJCC）的肿瘤-淋巴结-转移（tumor -node-metastasis, TNM）分期^[3]^，病理类型及分化程度按1999年世界卫生组织（World Health Organization, WHO）肺癌组织学类型分类标准且由病理科医师确认^[4]^。

### 1.2 分组

按血清白蛋白及尿素氮指标水平正常与否进行分组，正常值范围：白蛋白40.0 g/L-55.0 g/L、尿素3.30 mmol/L-8.22 mmol/L。指标水平正常定义为阴性，低于正常范围定义为阳性。

### 1.3 观察指标

分析血清白蛋白及尿素氮指标水平阴性与阳性两组的年龄、性别、病理学类型、临床分期、吸烟史、淋巴结转移及远处转移的临床特征的差异；并分别分析不同病理学类型中，血清白蛋白及尿素氮指标水平阴性与阳性两组的年龄、性别、临床分期、吸烟史、淋巴结转移及远处转移的临床特征及生存曲线的差异；并对有意义的临床特征进行分析，观察其对患者生存预后的影响。

### 1.4 统计学方法

采用SPSS 19.0软件进行统计学分析，采用卡方检验，生存分析采用*Kaplan*-*Meier*法并进行*Log*-*rank*检验，采用GraphPad Prism进行图片制作。*P*＜0.05为差异有统计学意义。

## 2 结果

### 2.1 一般情况

如表 1和表 2所示，本研究总共纳入原发性肺癌患者1, 098例，年龄19岁-90岁，平均60.65岁。其中男性757例（占69.0%），女性341例（31.0%）。纳入患者中，病理类型以腺癌为主，612例（55.8%）；鳞癌312例（28.4%），小细胞癌174例（15.8%）。临床分期中，早期肺癌（Ⅰ期-Ⅱ期）207例（18.9%），中晚期891例（81.1%）。此外，647例（58.9%）肺癌患者在诊断时伴有淋巴结转移，所有患者中有吸烟史的625例（56.9%）。

**1:** 血清白蛋白水平与1, 098例肺癌患者临床病理特征的关系 The relationship between serum albumin level and clinicopathologic features of 1, 098 lung cancer patients

Value	Negative (*n*=448)	Positive (*n*=650)	Total (*n*=1, 098)	*P*
Basic characteristics				
Age(yr)				＜0.001
＜45	42(9.4%)	48(7.4%)	90	
45-60	194(43.3%)	195(30.0%)	389	
＞60	212(47.3%)	407(62.6%)	619	
Gender				0.012
Male	290(64.7%)	467(71.8%)	757	
Female	158(35.3%)	183(28.2%)	341	
Histological classification				＜0.01
Squamous	98(21.8%)	214(32.9%)	312	
Adenocarcinoma	263(58.7%)	349(53.7%)	612	
SCLC	87(19.5%)	87(13.4%)	174	
Stages				0.001
Ⅰ	61(13.6%)	43(6.6%)	104	
Ⅱ	45(10%)	58(8.9%)	103	
Ⅲ	111(24.8%)	178(27.4%)	289	
Ⅳ	231(51.6%)	371(57.1%)	602	
Smoking status				0.172
No	204(45.5%)	269(41.4%)	473	
Yes	233(54.5%)	381(58.6%)	625	
Metastasis				
Brain				0.036
No	394(87.9%)	583(89.7%)	977	
Yes	54(12.2%)	67(10.3%)	121	
Bone				0.257
No	368(82.1%)	516(79.4%)	884	
Yes	80(17.9%)	134(20.6%)	214	
Liver				0.036
No	417(93.1%)	581(89.4%)	998	
Yes	31(6.9%)	69(10.4%)	100	
Adrenal gland				0.714
No	423(94.4%)	617(95.0%)	1, 040	
Yes	25(5.6%)	33(5.0%)	58	
Lymph node				0.135
No	196(48.7%)	255(39.2%)	451	
Yes	252(56.2%)	395(60.8%)	647	
Intrapulmonary				0.439
No	401(89.5%)	572(88.0%)	973	
Yes	47(10.5%)	78(12.0%)	125	
Pleural				0.025
No	396(88.4%)	543(83.5%)	939	
Yes	52(11.6%)	107(16.5%)	159	
Mediastinal				0.222
No	439(98.0%)	629(96.8%)	1, 068	
Yes	9(2.0%)	21(3.2%)	30	
SCLC: small cell lung cancer.				

**2:** 血尿素氮水平与1, 098例肺癌患者临床病理特征的关系 The relationship between serum urea level and clinicopathologic features of 1, 098 lung cancer patients

Value	Negative (*n*=643)	Positive (*n*=455)	Total (*n*=1, 098)	*P*
Basic characteristics				
Age(yr)				0.200
＜45	55(8.6%)	35(7.7%)	90	
45-60	240(37.3%)	149(32.7%)	389	
＞60	348(54.1%)	271(59.6%)	619	
Gender				0.823
Male	445(69.2%)	312(68.6%)	757	
Female	198(30.8%)	143(31.4%)	341	
Histological classification				0.925
Squamous	185(28.8%)	127(27.9%)	312	
Adenocarcinoma	358(55.7%)	254(55.8%)	612	
SCLC	100(15.5%)	74(16.3%)	174	
Stages				0.145
Ⅰ	58(9.0%)	46(10.1%)	104	
Ⅱ	55(8.6%)	48(10.5%)	103	
Ⅲ	185(28.8%)	104(22.9%)	289	
Ⅳ	345(53.6%)	257(56.5%)	602	
Smoking status				0.323
No	269(41.8%)	204(44.8%)	473	
Yes	374(58.2%)	251(55.2%)	625	
Metastasis				
Brain				0.576
No	575(89.4%)	402(88.4%)	977	
Yes	68(10.6%)	53(11.6%)	121	
Bone				0.608
No	521(81.0%)	363(79.8%)	884	
Yes	122(19.0%)	92(20.2%)	214	
Liver				0.331
No	589(91.6%)	409(89.9%)	998	
Yes	54(8.4%)	46(10.1%)	100	
Adrenal gland				0.791
No	610(94.9%)	430(95.5%)	1, 040	
Yes	33(5.1%)	25(5.5%)	58	
Lymph node				0.376
No	257(40.0%)	194(42.6%)	451	
Yes	386(60.0%)	261(57.4%)	647	
Intrapulmonary				0.355
No	565(87.9%)	408(89.7%)	973	
Yes	78(12.1%)	47(10.3%)	125	
Pleural				0.742
No	548(85.2%)	391(85.9%)	939	
Yes	95(14.8%)	64(14.1%)	159	
Mediastinal				0.831
No	626(97.4%)	442(97.1%)	1, 068	
Yes	17(2.6%)	13(2.9%)	30	

### 2.2 血清白蛋白及尿素氮水平与临床病理特征的关系

血清白蛋白水平阴性和阳性两组中，年龄、性别、病理学分型、肝转移、胸膜转移方面差异有统计学意义（*P*＜0.05），其余无统计学差异（*P*＞0.05）（表 1）。血尿素氮水平阴性和阳性两组均无统计学差异（*P*＞0.05）（表 2）。

### 2.3 肺癌不同病理分型中白蛋白水平与临床病理特征的关系

312例鳞癌患者，血清白蛋白水平阴性和阳性两组，年龄和肺内转移方面差异有统计学意义（*P*＜0.05）（表 3）。612例腺癌患者中，年龄、性别、临床分期及胸膜转移方面差异有统计学意义（*P*＜0.05）（表 4）。174例小细胞癌患者中各临床特征均无统计学差异（*P*＞0.05）（表 5）。

**3:** 血清白蛋白水平与312例鳞癌患者临床病理特征的关系 The relationship between serum albumin level and clinicopathologic features of 312 patients of squamous cell carcinoma

Value	Negative (*n*=98)	Positive (*n*=214)	Total (*n*=312)	*P*
Basic characteristics				
Age(yr)				＜0.001
＜45	5(5.1%)	5(2.3%)	10	
45-60	50(51.0%)	67(31.3%)	117	
＞60	38(43.9%)	14(66.4%)	185	
Gender				0.861
Male	89(90.8%)	193(90.2%)	282	
Female	158(35.3%)	183(28.2%)	30	
Stages				0.236
Ⅰ	13(13.3%)	15(7.0%)	28	
Ⅱ	15(15.3%)	26(12.1%)	41	
Ⅲ	33(33.7%)	84(39.3%)	117	
Ⅳ	37(37.7%)	89(41.6%)	126	
Smoking status				0.430
No	16(16.3%)	43(20.1%)	59	
Yes	82(83.7%)	171(79.9%)	253	
Metastasis				
Brain				0.751
No	91(92.9%)	207(96.7%)	298	
Yes	7(7.1%)	7(3.3%)	14	
Bone				0.751
No	86(87.8%)	185(86.4%)	271	
Yes	12(12.2%)	29(13.6%)	41	
Liver				0.200
No	93(94.9%)	194(90.7%)	287	
Yes	5(5.1%)	20(9.3%)	25	
Adrenal gland				0.424
No	92(93.9%)	207(96.7%)	299	
Yes	6(6.1%)	7(3.3%)	13	
Lymph node				0.303
No	45(45.9%)	85(39.7%)	130	
Yes	53(54.1%)	129(60.3%)	182	
Intrapulmonary				0.067
No	93(94.9%)	189(88.3%)	282	
Yes	5(5.1%)	25(11.7%)	30	
Pleural				0.641
No	90(91.8%)	193(90.2%)	283	
Yes	8(8.2%)	21(9.8%)	29	
Mediastinal				0.547
No	96(98.0%)	207(96.7%)	303	
Yes	2(2.0%)	7(3.3%)	9	

**4:** 血清白蛋白水平与612例腺癌患者临床病理特征的关系 The relationship between serum albumin level and clinicopathologic features of 612 patients of adenocarcinoma

Value	Negative (*n*=263)	Positive (*n*=349)	Total (*n*=612)	*P*
Basic characteristics				
Age(yr)				0.003
＜45	31(11.8%)	36(10.3%)	67	
45-60	101(38.4%)	92(26.4%)	193	
＞60	131(49.8%)	221(63.3%)	352	
Gender				0.021
Male	129(49.0%)	204(58.5%)	333	
Female	134(51.0%)	145(41.5%)	279	
Stages				0.001
Ⅰ	42(16.0%)	24(6.9%)	66	
Ⅱ	27(10.2%)	25(68%)	52	
Ⅲ	47(17.9%)	68(19.5%)	115	
Ⅳ	147(55.9%)	232(66.5%)	379	
Smoking status				0.234
No	164(62.4%)	21(57.6%)	365	
Yes	99(37.6%)	148(42.4%)	247	
Metastasis				
Brain				0.886
No	225(85.6%)	300(90.8%)	525	
Yes	38(14.4%)	49(14.0%)	87	
Bone				0.127
No	207(78.7%)	256(73.4%)	463	
Yes	56(21.3%)	93(26.6%)	149	
Liver				0.222
No	246(93.5%)	317(90.8%)	563	
Yes	17(6.5%)	32(9.2%)	49	
Adrenal gland				0.067
No	256(97.3%)	329(94.3%)	585	
Yes	7(2.7%)	20(5.7%)	27	
Lymph node				0.160
No	125(47.5%)	146(41.8%)	271	
Yes	138(52.5%)	203(58.2%)	341	
Intrapulmonary				0.673
No	226(85.9%)	304(87.1%)	530	
Yes	37(14.1%)	45(12.5%)	82	
Pleural				0.019
No	224(85.2%)	271(77.7%)	495	
Yes	39(14.8%)	78(22.3%)	117	
Mediastinal				0.069
No	260(98.9%)	337(96.6%)	597	
Yes	3(1.1%)	12(3.4%)	15	

**5:** 血清白蛋白水平与174例鳞癌患者临床病理特征的关系 The relationship between serum albumin level and clinicopathologic features of 174 small cell lung cancer patients

Value	Negative (*n*=87)	Positive (*n*=87)	Total (*n*=174)	*P*
Basic characteristics				
Age(yr)				0.567
＜45	6(6.9%)	7(8.0%)	13	
45-60	43(49.4%)	36(41.4%)	79	
＞60	38(43.7%)	44(50.6%)	82	
Gender				0.696
Male	72(82.8%)	70(80.5%)	142	
Female	15(17.2%)	17(19.5%)	32	
Stages				0.470
Ⅰ	6(6.9%)	4(4.6%)	10	
Ⅱ	3(3.4%)	7(8.0%)	10	
Ⅲ	31(35.6%)	26(29.9%)	57	
Ⅳ	47(54.1%)	50(57.5%)	97	
Smoking status				0.866
No	24(27.6%)	25(28.7%)	49	
Yes	63(72.4%)	62(71.3%)	125	
Metastasis				
Brain				0.886
No	225(85.6%)	300(90.8%)	525	
Yes	38(14.4%)	49(14.0%)	87	
Bone				0.635
No	78(89.7%)	76(87.4%)	154	
Yes	9(10.3%)	11(12.6%)	20	
Liver				0.089
No	78(89.7%)	70(80.5%)	148	
Yes	9(10.3%)	17(19.5%)	26	
Adrenal gland				0.135
No	75(86.2%)	81(93.1%)	156	
Yes	12(13.8%)	6(6.9%)	27	
Lymph node				0.738
No	26(29.9%)	24(27.6%)	50	
Yes	61(70.1%)	63(72.4%)	124	
Intrapulmonary				
No	82(94.3%)	79(90.8%)	161	0.387
Yes	5(5.7%)	8(9.2%)	13	
Pleural				
No	82(94.3%)	79(90.8%)	161	0.387
Yes	5(5.7%)	8(9.2%)	13	
Mediastinal				0.406
No	83(95.4%)	85(97.7%)	168	
Yes	4(4.6%)	2(2.3%)	6	

### 2.4 肺癌不同病理分型中血尿素氮水平与临床病理特征的关系

312例鳞癌患者（表 6）及612例腺癌患者中（表 7），尿素水平阴性和阳性两组中各临床特征均无统计学差异（*P*＞0.05）；174例小细胞癌患者中仅年龄差异有统计学意义（*P*＜0.05）（表 8）。

**6:** 血尿素氮水平与312例鳞癌患者临床病理特征的关系 The relationship between serum urea level and clinicopathologic features of 312 patients of squamous cell carcinoma

Value	Negative (*n*=185)	Positive (*n*=127)	Total (*n*=312)	*P*
Basic characteristics				
Age(yr)				0.396
＜45	8(4.3%)	2(1.6%)	10	
45-60	68(36.8%)	49(38.6%)	117	
＞60	109(58.9%)	76(59.8%)	185	
Gender				0.636
Male	166(89.7%)	116(91.3%)	282	
Female	19(10.3%)	11(8.7%)	30	
Stages				0.102
Ⅰ	17(9.2%)	11(8.7%)	28	
Ⅱ	18(9.7%)	23(18.1%)	41	
Ⅲ	77(41..6%)	40(31.5%)	117	
Ⅳ	73(39.5%)	53(41.7%)	126	
Smoking status				0.559
No	33(17.8%)	26(20.5%)	59	
Yes	152(82.2%)	62(79.5%)	253	
Metastasis				
Brain				0.344
No	175(94.6%)	106(83.5%)	298	
Yes	10(5.4%)	21(3.1%)	14	
Bone				0.141
No	165(89.2%)	106(83.5%)	271	
Yes	20(10.8%)	11(12.6%)	41	
Liver				0.727
No	171(92.4%)	116(913%)	287	
Yes	14(7.6%)	11(8.7%)	25	
Adrenal gland				0.456
No	176(95.1%)	123(96.9%)	299	
Yes	9(4.9%)	4(3.1%)	13	
Lymph node				0.340
No	73(39.5%)	57(44.9%)	130	
Yes	112(60.5%)	70(55.1%)	182	
Intrapulmonary				0.636
No	166(89.7%)	116(91.3%)	282	
Yes	19(10.3%)	11(8.7%)	30	
Pleural				0.750
No	167(90.3%)	116(91.3%)	283	
Yes	18(9.7%)	11(9.7%)	29	
Mediastinal				0.817
No	180(97.3%)	123(96.9%)	303	
Yes	5(2.7%)	4(3.1%)	9	

**7:** 血尿素氮水平与612例腺癌患者临床病理特征的关系 The relationship between serum urea level and clinicopathologic features of 612 patients of adenocarcinoma

Value	Negative (*n*=358)	Positive (*n*=254)	Total (*n*=612)	*P*
Basic characteristics				
Age(yr)				0.981
＜45	39(10.9%)	28(11.0%)	67	
45-60	114(31.8%)	79(31.1%)	193	
＞60	205(57.3%)	147(57.9%)	352	
Gender				0.843
Male	196(54.7%)	137(53.9%)	333	
Female	162(45.3%)	117(46.1%)	279	
Stages				0.272
Ⅰ	34(9.5%)	32(12.6%)	66	
Ⅱ	32(8.9%)	20(7.9%)	52	
Ⅲ	75(21.0%)	40(15.7%)	115	
Ⅳ	217(60.6%)	162(63.8%)	379	
Smoking status				0.800
No	212(59.2%)	153(60.2%)	365	
Yes	146(40.8%)	101(39.8%)	247	
Metastasis				
Brain				0.166
No	313(87.4%)	212(83.5%)	525	
Yes	45(12.6%)	42(16.5%)	87	
Bone				0.824
No	272(76.0%)	191(75.2%)	463	
Yes	86(24.0%)	63(24.8%)	49	
Liver				0.421
No	332(92.7%)	231(90.9%)	563	
Yes	26(7.3%)	17(9.1%)	49	
Adrenal gland				0.474
No	344(96.1%)	241(94.9%)	585	
Yes	14(3.9%)	13(5.1%)	27	
Lymph node				0.455
No	154(43.0%)	117(46.1%)	271	
Yes	204(57.0%)	137(53.9%)	341	
Intrapulmonary				0.331
No	306(85.5%)	224(88.2%)	530	
Yes	52(14.5%)	30(11.8%)	82	
Pleural				0.927
No	290(81.0%)	205(80.7%)	495	
Yes	68(19.0%)	49(19.3%)	117	
Mediastinal				0.516
No	348(97.2%)	249(98.0%)	597	
Yes	10(2.8%)	5(2.0%)	15	

**8:** 血尿素氮水平与174例鳞癌患者临床病理特征的关系 The relationship between serum urea level and clinicopathologic features of 174 small cell lung cancer patients

Value	Negative (*n*=100)	Positive (*n*=74)	Total (*n*=174)	*P*
Basic characteristics				
Age(yr)				＜0.001
＜45	8(8.0%)	5(6.7%)	13	
45-60	58(58.0%)	21(28.4%)	79	
＞60	34(34.0%)	48(64.9%)	82	
Gender				0.582
Male	83(83.0%)	59(79.7%)	142	
Female	17(17.0%)	15(20.3%)	32	
Stages				0.826
Ⅰ	7(7.0%)	3(4.1%)	10	
Ⅱ	5(5.0%)	5(6.7%)	10	
Ⅲ	33(33.0%)	24(32.4%)	57	
Ⅳ	55(55.0%)	42(56.8%)	97	
Smoking status				0.156
No	24(24.0%)	25(33.8%)	49	
Yes	76(76.0%)	49(66.2%)	125	
Metastasis				
Brain				0.469
No	87(87.0%)	67(90.5%)	154	
Yes	13(13.0%)	7(9.5%)	20	
Bone				0.469
No	84(84.0%)	66(89.2%)	154	
Yes	16(16.0%)	8(10.8%)	20	
Liver				0.685
No	86(86.0%)	62(83.8%)	148	
Yes	14(14.0%)	12(16.2%)	26	
Adrenal gland				0.862
No	90(90.0%)	66(89.2%)	156	
Yes	10(10.0%)	8(10.8%)	18	
Lymph node				0.668
No	30(30.0%)	20(27.0%)	50	
Yes	70(70.0%)	54(73.0%)	124	
Intrapulmonary				0.373
No	93(93.0%)	68(91.9%)	161	
Yes	7(7.0%)	6(8.1%)	13	
Pleural				0.373
No	91(91.0%)	70(94.6%)	161	
Yes	9(9%)	4(5.4%)	13	
Mediastinal				0.224
No	98(98.0%)	70(94.6%)	168	
Yes	2(2.0%)	4(5.4%)	6	

### 2.5 血清白蛋白水平与肺癌不同病理分型患者的预后

对这1, 098例患者进行24个月-36个月的随访，最终仅565例患者获得生存资料。在不同病理分型肺癌患者中，对血清白蛋白水平阴性及阳性组的患者进行生存分析。鳞癌患者，血清白蛋白阴性组与阳性组中位生存时间分别为36个月和19个月，前者中位生存期明显高于后者，差异有统计学意义（*P*＜0.05）（图 1A）。腺癌患者，中位生存时间分别为35个月和15个月，前者中位生存期明显高于后者，差异有统计学意义（*P*＜0.05）（图 1B）。小细胞癌患者中，两组差异无统计学意义（*P*=0.234）（图 1C）。

**1:**
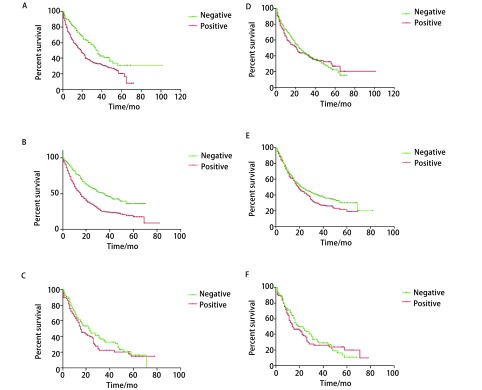
生存曲线。A：鳞癌患者血清白蛋白水平阴性及阳性组；B：腺癌患者血清白蛋白水平阴性及阳性组；C：小细胞癌患者血清白蛋白水平阴性及阳性组；D：鳞癌患者血尿素氮水平阴性及阳性组；E：腺癌患者血尿素氮水平阴性及阳性组；F：小细胞癌患者血尿素氮水平阴性及阳性组。 The *Kaplan*-*Meier* curves. A: Squamous cell carcinoma in negative and positive of serum albumin level; B: Adenocarcinoma in negative and positive of serum albumin level; C: Small cell lung cancer in negative and positive of serum albumin level; D: Squamous cell carcinoma in negative and positive of serum urea level; E: Adenocarcinoma in negative and positive of serum urea level; F: Small cell lung cancer in negative and positive of serum urea level.

### 2.6 血尿素氮水平与肺癌不同病理分型患者的预后

在不同病理分型肺癌患者中，对血尿素氮水平阴性及阳性组的患者进行生存分析。鳞癌患者、腺癌患者及小细胞癌患者中，两组差异均无统计学意义，*P*值分别为0.795（图 1D）、0.0629（图 1E）、0.204（图 1F）。

## 3 讨论

近年来，随着环境恶化和人口老龄化的加剧，各类呼吸系统疾病的发生不断增加，肺癌是发生于支气管黏膜上皮的常见的呼吸系统恶性肿瘤，已成为危害人类生命安全的主要疾病之一^[5]^。

血清白蛋白是血清总蛋白的主要蛋白质成分，由肝脏合成，是血浆中含量最丰富的蛋白质，他具有结合和运输内源性与外源性物质，维持血液胶体渗透压、营养支持等方面均起着重要作用，在生命过程中有着重要意义。血尿素氮则是人体蛋白质代谢的主要终末产物。本研究发现尿素氮与肺癌患者的临床特征及预后无明显统计学差异，但血清白蛋白水平则与肺癌的生存相关，白蛋白水平低的患者中位生存期明显低于白蛋白水平正常的患者。这可能与血清白蛋白可以反应患者的营养状况，而营养状况可影响患者预后。

恶性肿瘤疾病是一种消耗性疾病，肺癌患者长期处于消耗状态，导致体重降低、营养不良。血清白蛋白是作为评价营养的生化指标，是反映内脏蛋白水平的最佳和最简易的参数^[6]^。29个肿瘤研究中心的临床流行病学调查显示：白蛋白水平是肿瘤患者存活的预示因子^[7]^；60岁以上住院患者的死亡率与低白蛋白水平有关^[8]^。而白蛋白的含量也是评价机体免疫力的重要指标，当白蛋白含量＜30 g/L时，机体可能无法抵抗外来病原菌的侵袭，导致肺部感染等发生，尤其是接受手术或联合多种化疗药物治疗的患者，更易导致体内大量白蛋白的丢失。有研究^[9]^显示，当血清白蛋白＜30 g/L时，患者发生肺部感染概率显著增高，且为独立危险因素。有研究^[10]^表明，肺癌患者可存在血清白蛋白水平降低，且外科手术或者放化疗可进一步导致其血清白蛋白水平降低，因此白蛋白可能与肺癌患者的病情相关，可能用于其预后。此外，有研究^[11]^发现，血清总蛋白和白蛋白降低是肺癌患者发生深静脉血栓的危险因素之一，这也可能是导致肺癌患者生存期缩短的原因。肺癌患者血清白蛋白可作为其预后评价的参考指标。早期关注肺癌患者的营养状态，尤其是接受手术或是接受放化疗的患者，加强营养及蛋白质的摄入，可能提高其预后。但患者血清白蛋白水平以及预后均可能受高血压、糖尿病等基础疾病的影响，因此明确患者血清白蛋白水平对其预后的评估价值需更大样本量和更全面的研究。

综上所述，肺癌患者血清白蛋白可作为患者预后的一个预测指标。但由于其受多种因素影响，故临床上可以采用多种指标以及肿瘤标志物联合，更好地预测患者的预后。
